# Perceived weight discrimination in England: a population-based study of adults aged ⩾50 years

**DOI:** 10.1038/ijo.2014.186

**Published:** 2014-12-02

**Authors:** S E Jackson, A Steptoe, R J Beeken, H Croker, J Wardle

**Affiliations:** 1 Health Behaviour Research Centre, Department of Epidemiology and Public Health, University College London, London, UK; 2 Psychobiology Group, Department of Epidemiology and Public Health, University College London, London, UK

## Abstract

**Background:**

Despite a wealth of experimental studies on weight bias, little is known about weight discrimination at the population level. This study examined the prevalence and socio-demographic correlates of perceived weight discrimination in a large population-based sample of older adults.

**Methods:**

Data were from 5307 adults in the English Longitudinal Study of Ageing; a population-based cohort of men and women aged ⩾50 years. Weight discrimination was reported for five domains (less respect/courtesy; treated as less clever; poorer treatment in medical settings; poorer service in restaurants/stores; threatened/harassed) at wave 5 (2010–2011). Height and weight were measured at wave 4 (2008–2009). We used logistic regression to test the odds of weight discrimination in relation to weight status, age, sex, wealth, education and marital status.

**Results:**

Perceived weight discrimination in any domain was reported by 4.6% of participants, ranging from 0.8% in the normal-weight participants through 0.9, 6.7, 24.2 and 35.1% in individuals who were overweight or met criteria for class I, II and III obesity. Overall, and in each situation, odds of perceived weight discrimination were higher in younger and less wealthy individuals. There was no interaction between weight status and any socio-demographic variable. Relative to normal-weight participants, odds ratios for any perceived weight discrimination were 1.13 (95% confidence interval 0.53–2.40) in those who were overweight, 8.86 (4.65–16.88) in those with class I obesity, 35.06 (18.30–67.16) in class II obese and 56.43 (27.72–114.87) in class III obese.

**Conclusions:**

Our results indicate that rates of perceived weight discrimination are comparatively low in individuals who are overweight or have class I obesity, but for those with class II/III obesity, >10% had experienced discrimination in each domain, and >20% had been treated with less respect or courtesy. These findings have implications for public policy and highlight the need for effective interventions to promote equality.

## Introduction

The majority of adults living in the United Kingdom are overweight or obese. Recent statistics from the Health Survey for England indicate that >10 million adults living in England (25% of the adult population) are obese.^1^ However, despite the normative nature of carrying excess weight, an extensive literature documents weight bias and prejudiced attitudes towards people with obesity. People with obesity are stereotyped as lazy, less competent, lacking in self-discipline, non-compliant, sloppy and worthless.^2, 3, 4, 5^ Weight-related prejudice has been documented among health professionals, including doctors, nurses and psychologists,^2, 6^ and in employers and co-workers,^7^ teachers^8, 9^ and landlords,^10^ as well as peers,^11^ parents^7, 12^ and children.^13^ As a result, individuals with obesity are likely to be discriminated against—that is, treated unjustly or unequally—because of their weight. In the United States, weight discrimination has been identified as the fourth most prevalent form of discrimination, after gender, age and race discrimination.^14^

Research in this area has predominantly been based on case studies, experimental work and convenience or clinical samples, with few population-based studies. However, data from 2004 to 2006 on 1136 adults aged 35–74 years in the National Survey for Midlife Development in the United States (MIDUS) found lifetime experience of weight discrimination in any of the 11 situations (for example, not being promoted, being provided inferior medical care) was reported by 12.2% of the population, ranging from 3.9% of normal-weight individuals, 6.9% of overweight individuals and 14.2% of individuals with class I obesity (body mass index (BMI) 30–35 kg m^−^^2^) to 42.5% of individuals with class II or III obesity (BMI ⩾35 kg m^−^^2^), with rates of weight discrimination increasing more than any other form of discrimination since 1995–1996.^15^ In addition to weight status, associations between perceived weight discrimination and age, sex, ethnicity and socio-economic status (SES) were reported.^14^

MIDUS results have been very important in benchmarking the prevalence of weight discrimination in the US population. However, replication in other countries and cultures, where attitudes towards weight might be different, is important.^16^ In order to add to previous results and enhance current knowledge, the first objective of the present study was therefore to examine the extent of perceived weight discrimination in a large population-based sample of middle-aged and older adults, assessed as part of the English Longitudinal Study of Ageing (ELSA). Although the question wording was not identical, this allowed broad replication of MIDUS results in an English population.

Weight discrimination can take various forms and occurs across a range of domains, from being treated disrespectfully in everyday life to receiving poorer treatment in medical settings. MIDUS assessed discrimination across 11 situations, but the results of multivariate analyses for the individual types of discrimination were not presented.^14, 15^ A secondary objective of our study was therefore to explore the prevalence and correlates of weight discrimination across five domains.

To summarise, the aim of the present study was to examine the prevalence and patterns of perceived weight discrimination in five domains by weight status and socio-demographic characteristics, in a large population-based sample of middle-aged and older adults living in England. We hypothesised that perceived weight discrimination would be more common among individuals who were heavier, younger, female and from lower SES backgrounds.

## Materials and Methods

### Study population

Details on the ELSA cohort and sampling method have been published elsewhere,^17^ but briefly, ELSA is a panel study recruited from households with one or more members aged ⩾50 years responding to the Health Survey for England in 1998, 1999 and 2001 (core sample: *n*=12 099), with ‘refreshment samples’ added from additional rounds of the Health Survey for England in 2006, 2008 and 2012. Comparisons of the socio-demographic characteristics of ELSA participants against results from the national census indicate that the sample is broadly representative of the English population.^17^ Since 2002, participants have been interviewed in biennial waves where they do a computer-assisted personal interview and complete self-administered questionnaires. In alternate (even-numbered) waves, a nurse visits the home to carry out a health examination that includes anthropometry. In the wave 5 interview (2010–2011), participants were asked about their experience of discrimination across a range of areas, including weight. The present analyses use these data plus measured anthropometric data from wave 4 (2008–2009). We restricted our sample to participants who had both answered the questions on discrimination and had data on BMI at wave 4 (*n*=5350). As discrimination questions were reliant on memory of recent experiences, we excluded 43 participants with doctor-diagnosed dementia, leaving a final analytical sample of 5307 men and women. ELSA has received approval from various ethics committees, including the London Multi-Centre Research Ethics Committee, and full informed consent has been obtained from all participants. Data are publicly available at http://discover.ukdataservice.ac.uk.

### Measures

In previous population studies, weight discrimination has variously been found to be related to age, sex and SES as defined by the level of education or occupation.^14, 15, 18, 19^ We therefore explored associations with age, sex, education and household wealth (a sensitive indicator of SES in this age group). Education and wealth are both indicators of socioeconomic position, and might therefore be expected to be highly correlated, but we felt it was important to include both variables because previous studies have found them to be associated with perceived discrimination in different ways, with less wealthy and more highly educated individuals being more likely to report discrimination.^20, 21, 22, 23^ We also included marital status in our analyses, as a study of weight-loss programme participants found that spouses are a common source of weight-related mistreatment.^24^

#### Perceived weight discrimination

Questions on perceived discrimination were based on items developed and used widely in other longitudinal studies in the United States of America, notably MIDUS and the Health and Retirement Study.^14, 15, 25, 26, 27, 28^ Participants were asked how often they had encountered five discriminatory situations: ‘*In your day-to-day life, how often have any of the following things happened to you: 1) you are treated with less respect or courtesy; 2) you receive poorer service than other people in restaurants and stores; 3) people act as if they think you are not clever; 4) you are threatened or harassed; 5) you receive poorer service or treatment than other people from doctors or hospitals.*’ Possible response options were almost every day, at least once a week, a few times a month, a few times a year, less than once a year or never. We dichotomised responses to indicate whether or not respondents had ever experienced discrimination in each situation (never vs all other options). A follow-up question asked participants to indicate whether they perceived the discrimination to be due to each of a list of options, including weight, age, gender, race, physical disability and sexual orientation. Participants could attribute more than one reason to their experiences of discrimination. Those who attributed any experience of discrimination to their weight are treated in our study as cases of perceived weight discrimination. Where individuals reported weight discrimination, we looked both at individual domains and created an overall score reflecting any experiences of weight discrimination (weight discrimination in at least one domain vs no reported weight discrimination in any domain) for comparison with MIDUS results.

#### Anthropometry

Weight was measured to the nearest 0.1 kg using Tanita THD-305 portable electronic scales (Tanita, Amsterdam, the Netherlands) and height to the nearest millimeter using a portable stadiometer (Seca, Birmingham, UK), from which BMI was calculated. Nurses also recorded any factors that could compromise measurement reliability (for example, participant was stooped or unwilling to remove shoes). Anthropometric data judged by the nurse to be unreliable were excluded. We classified BMI according to the World Health Organisation cutoffs as underweight (<18.5 kg m^−^^2^), normal-weight (18.5–24.9 kg m^−^^2^), overweight (25–29.9 kg m^−^^2^), obese class I (30–34.9 kg m^−^^2^), obese class II (35–39.9 kg m^−^^2^) and obese class III (⩾40 kg m^−^^2^).^29^ Because of the small number of underweight participants (*n*=38, 0.7%), they were included in the normal-weight group for all analyses.

#### Socio-demographic information

Interviewers collected information on age, sex, wealth, the highest level of education and marital status. For these analyses, age was grouped into 50–59, 60–69, 70–79 and ⩾80 years. Total wealth (excluding regular pension payments but including lump sums from private pension that have already been received but not yet consumed) was defined as financial wealth, physical wealth (such as business wealth, land or jewels) and housing wealth (primary and secondary residential housing wealth) minus debts. Wealth was categorised into five equal groups of net total non-pension wealth measured at the benefit unit level (a benefit unit is a couple or single person along with any dependent children they might have) across all ELSA participants who took part in wave 5. Wealth has been identified as a particularly appropriate indicator of SES in this age group.^30^ We classified education as low (no formal qualifications), intermediate (Certificate of Secondary Education or equivalent) or high (A levels or equivalent through to higher degrees). Marital status was categorised as married, never married, divorced or widowed.

### Statistical analysis

Analyses were performed using SPSS version 20 (IBM, New York, NY, USA), with a *P*-value <0.05 determining statistical significance. Our primary outcome was the perception of weight discrimination in any of the five discriminatory situations, and secondary outcomes were perceived weight discrimination in each different discriminatory situation. For comparison, we also assessed the prevalence of perceived age, sex, race, physical disability and sexual orientation discrimination. Logistic regression was used to examine associations between perceived weight discrimination and weight status, age, sex, wealth, education and marital status. We ran univariate models to test relationships with each individual covariate and a multivariable model to assess the independent contributions of each covariate in predicting weight discrimination. Because certain socio-demographic groups may be more or less likely to attribute experiences of discrimination to their weight according to how they perceive their own weight (for example, women tend to be more likely than men to perceive themselves as overweight and feel more pressure to conform to societal body weight ideals^31, 32^ and, as a result, may be more likely to attribute any experiences of discrimination to their weight), we tested for interactions between weight status and all socio-demographic variables (age, sex, wealth, education and marital status).

## Results

Table 1 shows the number of participants in each socio-demographic and weight group. The mean age of the sample was 67.11 (s.d. 8.88) years, with 41.3% of participants in their 60s. Most participants were overweight (41.4%) or obese (class I: 21.7% class II: 6.9% class III: 2.5%), with only 27.4% in the normal-weight range. Obesity prevalence by age group was 33.8% in those aged 50–59 years, 31.5% in those aged 60–69 years, 30.5% in those aged 70–79 years and 25.8% in those aged ⩾80 years. The sample included more women than men (55.7% vs 44.3%). Around two-thirds (67.5%) were married, and almost half (45.7%) were highly educated. Cases for this analysis were also slightly wealthier than the general ELSA sample, with the upper quintiles of wealth slightly overrepresented (23.3% in the highest quintile and 21.8% in the second highest) and the lowest quintile underrepresented (15.7%).

Perceived weight discrimination in any situation was reported by 4.6% of the full sample, with substantial variation by weight status, increasing from 0.8% in normal-weight individuals and 0.9% in individuals who were overweight to 6.7% in those with class I obesity, 24.2% in those with class II obesity and 35.1% in those with class III obesity. Compared with other forms of discrimination, weight discrimination was jointly the third most prevalent, alongside physical disability discrimination (4.6%), behind discrimination on the basis of age (39.5%) and sex (11.1%) and ahead of discrimination on the basis of race (1.6%) and sexual orientation (0.7%).

Unadjusted analyses showed significant associations between perceived weight discrimination and weight status, age, sex and wealth (Table 1), with heavier, younger, female and less wealthy individuals more likely to report weight discrimination. When all covariates were entered into a multivariable model, the association with sex was no longer significant, principally due to inclusion of weight status in the model, because more women than men had severe obesity (class II: 7.9% vs 5.7% class III: 3.5% vs 1.3%). Education and marital status were not associated with perceived weight discrimination in unadjusted models, but in the multivariable model an intermediate level of education was associated with higher odds of perceived weight discrimination than a low level of education.

There were no significant interactions between weight status and any other covariate, indicating that obesity was equally strongly associated with perceived weight discrimination across all the socio-demographic groups.

Analyses of the different discriminatory situations showed consistent patterns of results. Overall prevalence of perceived weight discrimination ranged from 1.7% for receiving poorer service or treatment in a medical setting to 4.3% for being treated with less respect or courtesy (Table 2). The majority (90.6%) of participants reporting weight discrimination had experienced discrimination in more than one situation (mean number of situations=3.23, s.d.=1.23), with 17.6% reporting experiences of discrimination in all the five situations. Weight status was the strongest correlate of weight discrimination in each situation (Table 3), with a particularly marked increase in perceived weight discrimination in participants with class II/III obesity. There were also consistent inverse associations with age and wealth across all discriminatory situations, although the association with age was the least pronounced in medical settings. Individuals with higher levels of education were more likely than those with a low level to report receiving poorer service in restaurants or stores because of their weight (intermediate: odds ratio=1.76, 95% confidence interval (CI)=1.09–2.83; higher=1.53, 95% CI=0.94–2.51), and there were non-significant trends in the same direction for being threatened or harassed and being treated with less courtesy. Sex and marital status were not significantly related to perceived weight discrimination in any specific situation after adjusting for covariates.

## Discussion

This is the first population-based study of weight discrimination in middle-aged and older people in the United Kingdom. In this sample of adults aged ⩾50 years, 4.6% reported experiences of weight discrimination. Prevalence was effectively zero in individuals who were normal weight or overweight (<1%), and still relatively uncommon among those with class I obesity (6.7%), but rose to 24.2% and 35.1% in those with class II and III obesity. Among individuals with obesity, reports of weight discrimination varied across the five discriminatory situations assessed, with the most frequent being treated with less respect or courtesy (12.0%) and the least frequent receiving poorer treatment in medical settings (4.7%). Nonetheless, among those with class II or III obesity, >10% felt they received poorer treatment in medical settings. There were inverse associations with age and wealth, with younger and less wealthy individuals more likely to report weight discrimination. Most people who reported weight discrimination had experienced discrimination across multiple situations. Correlates were consistent across the different discriminatory situations, with weight status, age and wealth most strongly related to odds of weight discrimination in each situation.

Despite differences in measures of discrimination, the observed prevalence of perceived weight discrimination in this sample of English middle-aged and older adults was very similar to results from MIDUS, where rates were 5.3% in 55–64-year-olds and 4.0% in 65–74-year-olds.^14^ Extrapolating our findings using data on population size and obesity prevalence from the 2010 Health Survey for England,^33^ we estimate that 646 882 middle-aged and older adults (⩾55 years) in England are currently affected by weight discrimination. Our findings confirm previous results from the United States showing a decline in weight discrimination with age^14, 15^ and extend them by demonstrating that the decline continues to people in their 70s and 80s. The inverse association between age and perceived weight discrimination might reflect a decline in social expectation of thinness with ageing or an increase in the salience of other causes of discrimination (for example, age discrimination^21^).

Particularly high levels of weight discrimination were observed among individuals with class II or III obesity, among whom 1 in 10 reported receiving poorer service or treatment than other people from doctors or hospitals. Perceived weight discrimination in primary care has important implications for the health of patients with obesity, who may delay or avoid essential medical care because of their weight.^34, 35, 36^ As older people use health services most frequently, perceived weight discrimination in medical settings might be particularly important. Also interesting is that in contrast to the previous North American studies which observed an elevated risk of weight discrimination among overweight individuals,^14, 15^ in the present sample, weight discrimination was almost exclusively restricted to participants with obesity, with no excess risk of perceived discrimination in those who were overweight compared with normal-weight participants. This may be due to the different age range, different population mix in the United Kingdom or a different culture, but it could also reflect normative shifts in body size between the time of MIDUS data collection (1995–1996 and 2004–2006) and wave 5 of ELSA (2010–2011), so that overweight is the ‘new normal’.^37^

We observed a higher prevalence of perceived weight discrimination in women than men (5.2% vs 3.8%), but this difference was explained by the higher rates of obesity among women in our sample. In the MIDUS cohort,^14^ the sex difference was more pronounced (10.3% in women vs 4.9% in men) and persisted after adjustment for weight status. However, prevalence grew more similar between men and women with increasing age, which might be why we observed a smaller sex difference in our sample, who were all aged ⩾50 years.

In line with previous research,^14^ individuals with higher levels of education were more likely than those who were less educated to report experiences of weight discrimination. This finding may seem counterintuitive given that increasing wealth, another indicator of SES, was associated with lower prevalence of weight discrimination. However, a similar pattern of results has been observed in previous studies of perceived discrimination.^18, 21, 22, 23^ More educated people may have a greater propensity to recognise inequities,^22^ whereas wealthier individuals may be less likely to experience discrimination due to reduced exposure to discriminatory situations or a greater sense of control or security.^21^ However, it is important to note that the association between education and weight discrimination was one of a considerable number of comparisons, and the risk of perceiving weight discrimination did not increase linearly with increasing levels of education, so it could be a chance finding. Further investigation is needed to clarify associations between perceived weight discrimination and different indicators of SES.

Weight discrimination is often justified on the basis that it might encourage people with obesity to lose weight,^38^ but current evidence suggests that those who experience weight discrimination are actually more likely to engage in obesity-promoting behaviours, including problematic eating,^39, 40, 41, 42^ refusal to diet^7, 43^ and avoidance of physical activity,^44, 45^ and may be more likely to gain weight.^46^ Weight discrimination is also associated with poorer wellbeing,^4^ depression,^41, 47, 48^ lower self-esteem and self-acceptance^28, 41^ and body image dissatisfaction.^41, 45, 49^ We cannot be certain that the effects are causal in a cohort design, but insofar as there is any causal effect from discrimination to wellbeing, tackling weight discrimination could improve the health and wellbeing for a significant proportion of individuals with obesity.

In the United Kingdom, the Equality Act 2010 legally protects individuals from discrimination on the basis of age, sex, race, disability, religion or beliefs, sexual orientation, marital status, pregnancy or gender reassignment, but there are currently no laws prohibiting weight discrimination. When we compared the prevalence of weight discrimination with other types of discrimination, it ranked as the third most prevalent after age and sex discrimination, was ahead of race and sexual orientation discrimination and was reported as commonly as discrimination on the basis of physical disability. Findings from the United States suggest that the introduction of anti-discrimination laws could potentially help to reduce weight discrimination. Only one US state (Michigan) currently legislates against weight discrimination, explicitly stating that it is illegal to discriminate based on an applicant’s or employee’s weight, and the prevalence of weight discrimination in the workplace is substantially lower than in other US states.^50^ Recent surveys have indicated increasing support in the general population for introduction of laws against weight discrimination, with 69% supportive of extending disability protections to individuals with obesity, 76% supporting the inclusion of body weight as a protected category in civil rights laws and 79% expressing support for laws prohibiting weight discrimination in the workplace.^51^

This study had a number of limitations. The cross-sectional design does not allow us to draw conclusions on causality. It is not possible to establish whether heavier individuals were more likely to experience weight discrimination or more likely to attribute discrimination they experienced to their weight. Weight discrimination was determined by self-reports of past experiences, hence may be subject to recall bias. Although we excluded participants known to have dementia, recall bias may be particularly prominent among participants at the older end of the spectrum, which may have led us to underestimate prevalence of weight discrimination in the oldest age groups. Weight was not measured in the same data collection wave as discrimination, and participants may have changed weight prior to reporting discrimination. If weight loss was associated with a decline in perceived weight discrimination, this may have led to underestimates of the prevalence of weight discrimination among the heavier weight categories. It was possible to attribute multiple reasons to experiences of discrimination, so they are not necessarily limited to weight discrimination; although by asking about a range of potential reasons for discrimination, weight was not the obvious focus, which may have helped to avoid bias among respondents with overweight or obesity. The discrimination questions asked about five situations, but there are others that may be particularly relevant to weight discrimination, for example, being viewed unfavourably as a potential romantic partner or having to pay more on public transport for occupying two passenger seats. By omitting these, the prevalence of weight discrimination may again have been underestimated. The ELSA sample comprises predominantly white, older adults, and experiences or perceptions of weight discrimination may be different in younger adults or different ethnic groups. Given that we found an inverse association with age, it is likely that rates would be higher among the younger groups. In addition, our analyses were restricted to ELSA participants who had data on discrimination and BMI. In line with retention in other longitudinal studies,^52^ the analysed sample was slightly younger, wealthier and less overweight than the total ELSA sample (although the level of perceived weight discrimination did not differ), so results may not be population-representative. It was not possible to examine differences in perceived weight discrimination by ethnicity in the present sample due to the small number of participants from minority groups (*n*=112, 2.1%). Data from MIDUS indicate higher rates of weight discrimination among the non-white ethnic groups in the United States,^14^ although it is not clear whether this is due to differences in body weight. Evidence of a race–sex interaction on perceived weight discrimination has also been reported.^19^ Further research is needed to establish whether similar ethnic differences in perceived weight discrimination exist in the United Kingdom.

These results indicate that a substantial proportion of middle-aged and older adults with obesity reported being affected by weight discrimination in their everyday lives. Although further investigation is required in order to fully understand the impact of weight discrimination at an individual and societal level, these findings have implications for public policy and highlight the need for effective interventions to promote equality.

## Figures and Tables

**Table 1:** Prevalence of, and univariate and multivariable logistic regression models predicting, any perceived weight discrimination in the English Longitudinal Study of Ageing

	n	*PWD (%)*	*Unadjusted OR (95% CI)*	P	*Adjusted OR (95% CI)*	P
Overall	5307	4.6	—	—	—	—
						
*Weight status (*n*=5307)*						
Normal-weight	1456	0.8	1.00	—	1.00	—
Overweight	2197	0.9	1.21 (0.58–2.53)	0.618	1.13 (0.53–2.40)	0.759
Obese class I	1152	6.7	9.41 (4.98–17.79)	<0.001	8.86 (4.65–16.88)	<0.001
Obese class II	368	24.2	41.91 (22.11–79.42)	<0.001	35.06 (18.30–67.16)	<0.001
Obese class III	134	35.1	70.97 (35.55–141.66)	<0.001	56.43 (27.72–114.87)	<0.001
						
*Age in years (*n*=5307)*						
50–59	1167	8.9	1.00	—	1.00	—
60–69	2191	4.4	0.47 (0.36–0.63)	<0.001	0.50 (0.36–0.70)	<0.001
70–79	1425	2.5	0.27 (0.18–0.39)	<0.001	0.30 (0.19–0.48)	<0.001
⩾80	524	1.3	0.14 (0.06–0.30)	<0.001	0.18 (0.08–0.43)	<0.001
						
*Sex (*n*=5307)*						
Male	2349	3.8	1.00	—	1.00	—
Female	2958	5.2	1.38 (1.06–1.80)	0.018	0.99 (0.73–1.35)	0.943
						
*Wealth (*n*=5014)*						
1 (lowest)	787	7.9	1.00	—	1.00	—
2	971	6.7	0.84 (0.59–1.20)	0.341	0.91 (0.60–1.40)	0.676
3	993	3.8	0.47 (0.31–0.71)	<0.001	0.51 (0.32–0.83)	0.006
4	1095	3.4	0.41 (0.27–0.62)	<0.001	0.55 (0.33–0.89)	0.015
5 (highest)	1168	2.5	0.30 (0.19–0.47)	<0.001	0.49 (0.29–0.83)	0.008
						
*Education (*n*=5299)*						
Low	1225	4.2	1.00	—	1.00	—
Intermediate	1651	5.6	1.35 (0.95–1.91)	0.094	1.51 (1.01–2.27)	0.045
High	2423	4.1	0.96 (0.68–1.35)	0.820	1.29 (0.85–1.95)	0.238
						
*Marital status (*n*=5306)*						
Married	3582	4.7	1.00	—	1.00	—
Never married	283	4.6	0.97 (0.55–1.73)	0.924	0.67 (0.35–1.31)	0.244
Divorced	639	5.5	1.17 (0.81–1.70)	0.410	0.81 (0.51–1.26)	0.344
Widowed	802	3.4	0.70 (0.47–1.06)	0.096	0.96 (0.59–1.57)	0.869

Abbreviations: CI, confidence interval; OR, odds ratio; PWD, perceived weight discrimination.

Adjusted odds ratios are adjusted for weight status, age, sex, wealth, education and marital status.

**Table 2:** Number and proportion of participants reporting any perceived weight discrimination in different discriminatory situations (*N*=5307)

	*Less courtesy*		*Less clever*		*Service setting*		*Harassed*		*Medical setting*	
	n	*%*	n	*%*	n	*%*	n	*%*	n	*%*
Overall	227	4.3	185	3.5	176	3.3	112	2.1	89	1.7
										
*Weight status* (n=*5307*)										
Normal-weight	10	0.7	10	0.7	7	0.5	5	0.3	5	0.3
Overweight	19	0.9	15	0.7	15	0.7	12	0.5	6	0.3
Obese class I	72	6.3	62	5.4	56	4.9	36	3.1	24	2.1
Obese class II	86	23.4	68	18.5	67	18.2	40	10.9	37	10.1
Obese class III	40	29.9	30	22.4	31	23.1	19	14.2	17	12.7
										
*Age in years* (n=*5307*)										
50–59	101	8.7	85	7.3	74	6.3	57	4.9	40	3.4
60–69	91	4.2	65	3.0	69	3.1	41	1.9	32	1.5
70–79	28	2.0	31	2.2	28	2.0	12	0.8	13	0.9
⩾80	7	1.3	4	0.8	5	1.0	2	0.4	4	0.8
										
*Sex* (n=*5307*)										
Male	85	3.6	69	2.9	59	2.5	44	1.9	27	1.1
Female	142	4.8	116	3.9	117	4.0	68	2.3	62	2.1
										
*Wealth* (n=*5014*)										
1 (lowest)	58	7.4	47	6.0	43	5.5	26	3.3	26	3.3
2	59	6.1	49	5.0	43	4.4	33	3.4	23	2.4
3	36	3.6	27	2.7	27	2.7	13	1.3	15	1.5
4	35	3.2	31	2.8	30	2.7	20	1.8	13	1.2
5 (highest)	26	2.2	20	1.7	21	1.8	12	1.0	4	0.3
										
*Education* (n=*5299*)										
Low	46	3.8	44	3.6	34	2.8	18	1.5	19	1.6
Intermediate	85	5.1	69	4.2	68	4.1	43	2.6	34	2.1
High	96	4.0	72	3.0	74	3.1	51	2.1	36	1.5

*Marital status* (n=*5306*)										
Married	160	4.5	128	3.6	118	3.3	81	2.3	58	1.6
Never married	13	4.6	9	3.2	11	3.9	4	1.4	2	0.7
Divorced	29	4.5	25	3.9	25	3.9	17	2.7	15	2.3
Widowed	25	3.1	23	2.9	22	2.7	10	1.2	14	1.7

**Table 3:** Multivariable logistic regression models showing socio-demographic associations with reporting perceived weight discrimination in different discriminatory situations (*N*=5307)

	*Less courtesy*		*Less clever*		*Service setting*		*Harassed*		*Medical setting*	
	*OR (95% CI)*	P	*OR (95% CI)*	P	*OR (95% CI)*	P	*OR (95% CI)*	P	*OR (95% CI)*	P
*Weight status*										
Normal-weight	1.00	—	1.00	—	1.00	—	1.00	—	1.00	—
Overweight	1.17 (0.53–2.57)	0.696	0.95 (0.42–2.16)	0.910	1.34 (0.53–3.39)	0.532	1.36 (0.46–4.01)	0.576	0.68 (0.20–2.35)	0.539
Obese class I	9.04 (4.61–17.75)	<0.001	7.57 (3.83–14.98)	<0.001	10.14 (4.56–22.52)	<0.001	8.73 (3.37–22.60)	<0.001	4.98 (1.86–13.34)	0.001
Obese class II	36.77 (18.66–72.46)	<0.001	26.42 (13.28–52.55)	<0.001	39.64 (17.83–88.10)	<0.001	29.00 (11.20–75.10)	<0.001	23.33 (8.96–60.76)	<0.001
Obese class III	47.20 (22.39–99.49)	<0.001	30.60 (14.19–65.97)	<0.001	48.46 (20.40–115.11)	<0.001	37.67 (13.43–105.70)	<0.001	26.79 (9.44–76.02)	<0.001
										
*Age in years*										
50–59	1.00	—	1.00	—	1.00	—	1.00	—	1.00	—
60–69	0.48 (0.34–0.68)	<0.001	0.40 (0.28–0.58)	<0.001	0.54 (0.37–0.79)	0.002	0.44 (0.28–0.69)	<0.001	0.56 (0.33–0.95)	0.031
70–79	0.24 (0.15–0.39)	<0.001	0.30 (0.19–0.49)	<0.001	0.37 (0.22–0.62)	<0.001	0.22 (0.11–0.45)	<0.001	0.35 (0.17–0.72)	0.005
⩾80	0.18 (0.08–0.43)	<0.001	0.11 (0.04–0.32)	<0.001	0.20 (0.08–0.55)	0.002	0.12 (0.03–0.52)	0.005	0.31 (0.10–0.99)	0.048
										
*Sex*										
Male	1.00	—	1.00	—	1.00	—	1.00	—	1.00	—
Female	0.98 (0.71–1.35)	0.895	0.95 (0.67–1.34)	0.749	1.21 (0.85–1.74)	0.297	0.89 (0.58–1.38)	0.612	1.23 (0.74–2.04)	0.430
										
*Wealth*										
1 (lowest)	1.00	—	1.00	—	1.00	—	1.00	—	1.00	—
2	0.82 (0.53–1.27)	0.369	0.89 (0.56–1.42)	0.622	0.86 (0.53–1.40)	0.548	1.08 (0.60–1.94)	0.792	0.77 (0.41–1.45)	0.421
3	0.49 (0.30–0.79)	0.004	0.50 (0.29–0.86)	0.011	0.56 (0.32–0.97)	0.037	0.42 (0.20–0.87)	0.020	0.54 (0.27–1.10)	0.092
4	0.51 (0.31–0.84)	0.009	0.64 (0.38–1.09)	0.103	0.68 (0.40–1.18)	0.168	0.69 (0.35–1.35)	0.277	0.50 (0.23–1.05)	0.067
5 (highest)	0.42 (0.24–0.72)	0.002	0.45 (0.25–0.83)	0.011	0.54 (0.29–0.99)	0.046	0.44 (0.20–0.95)	0.037	0.17 (0.05–0.51)	0.002
										
*Education*										
Low	1.00	—	1.00	—	1.00	—	1.00	—	1.00	—
Intermediate	1.53 (1.00–2.34)	0.051	1.26 (0.81–1.96)	0.303	1.76 (1.09–2.83)	0.021	1.78 (0.96–3.28)	0.067	1.59 (0.83–3.03)	0.161
High	1.41 (0.91–2.17)	0.124	1.04 (0.66–1.65)	0.861	1.53 (0.94–2.51)	0.087	1.78 (0.96–3.32)	0.069	1.74 (0.91–3.35)	0.096
										
*Marital status*										
Married	1.00	—	1.00	—	1.00	—	1.00	—	1.00	—
Never married	0.71 (0.36–1.38)	0.307	0.60 (0.28–1.31)	0.203	0.92 (0.45–1.88)	0.809	0.45 (0.16–1.31)	0.145	0.32 (0.08–1.38)	0.126
Divorced	0.66 (0.41–1.07)	0.094	0.74 (0.45–1.23)	0.246	0.89 (0.54–1.49)	0.664	0.86 (0.47–1.57)	0.616	1.00 (0.52–1.94)	0.999
Widowed	1.01 (0.60–1.67)	0.984	1.19 (0.70–2.01)	0.528	1.16 (0.68–1.99)	0.593	0.97 (0.46–2.04)	0.933	1.43 (0.71–2.89)	0.323

Abbreviations: CI, confidence interval; OR, odds ratio.

Odds ratios are adjusted for weight status, age, sex, wealth, education and marital status.
